# Extreme original data yield extreme decline effects

**DOI:** 10.1371/journal.pbio.3001996

**Published:** 2023-02-06

**Authors:** Jeff C. Clements, Josefin Sundin, Timothy D. Clark, Fredrik Jutfelt

**Affiliations:** 1 Department of Biology, Norwegian University of Science and Technology, Trondheim, Norway; 2 Department of Aquatic Resources, Swedish University of Agricultural Sciences, Drottningholm, Sweden; 3 School of Life and Environmental Sciences, Deakin University, Geelong, Australia; 4 Department of Biological and Environmental Sciences, University of Gothenburg, Gothenburg, Sweden

## Abstract

Clements et al. respond to Munday’s claim that his “reanalysis shows there is not an extreme decline effect in fish ocean acidification studies”. They contend that extreme data reported in early studies authored by Dixson and Munday indeed result in an “extreme” decline effect in this field, and conclude that the decline effect is primarily driven by papers by particular authors.

Our meta-analysis showing a dramatic decline effect in ocean acidification (OA) studies on fish behavior [1] was criticized and reanalyzed by Dr. Munday [2]. After applying changes to the dataset in a seemingly biased direction (see Supp. Data 1 at https://osf.io/7spzx/), Munday found a slightly less “extreme” decline effect than the original. Nonetheless, Munday’s reanalyzed decline effect remains one of the strongest examples of this phenomenon in ecology [3], despite the claim that his “*reanalysis shows there is not an extreme decline effect in fish ocean acidification studies*.”

Why is the decline effect still present in the reanalysis, and why did early studies have such inflated effect sizes? The reason is that many early papers by Munday and colleagues included data that are extreme in and of themselves, and likely nonbiological in origin [4].

Alongside specific comments and revisions to our dataset based on subjective criteria (some we accept, many we do not; Supp. Data 1, Supp. Table 1 at https://osf.io/7spzx/), Munday critiques how we assigned values to means of zero when computing effect sizes. Since biological data rarely have means and variances of precisely zero, treating means of zero in meta-analyses has not been extensively explored. We agree with Munday that a discussion about how to handle mean values of zero in calculations of effect size is warranted, which we elaborate upon in **Box 1**. Munday’s critique is that our use of small fractional means for percentage and proportional data where the original means and variances are precisely zero “artificially inflates” effect size estimates. But context matters—the “artificially inflated” effect sizes in early studies are derived from initial data that are highly unlikely to begin with. For example, in a paper by Dr. Dixson and colleagues [5], choice-flume experiments measuring time spent in predator chemical cues yielded means for control and OA-exposed fish of precisely 0% and 100%, respectively, each with variances of exactly 0%.

**Box 1. Assigning values to zero and near-zero means and variances**

The main aspect of Munday’s revised dataset that reduces the magnitude of the decline effect reported in our original analysis is the prescription of values to zero means. We agree that effect sizes can be difficult to estimate for percentage data where one treatment approaches 100% and the other approaches 0%. In panels G-H of Fig 2 in [2], Munday reanalyzes our dataset by replacing our zero-mean values of 0.0001% for percentage data with values of 1% (0.01 for proportion data). With respect to these particular values presented in Munday’s Fig 2, using 1% is an inaccurate representation of 0%, as the percentages in the actual data were not 1%, they were 0% with no variance. In these papers, 0% is not derived from a single measurement but is the result of multiple consecutive measurements on individual fish that all recorded 0% time in the chemical cue side of a choice-flume. Hence, we can be very certain that the actual number is 0%. As the variance around the 0% mean is also zero, the precision of the mean is interpreted to be very high and confidence in the real mean value being 0% is similarly high. From a Bayesian perspective, confidence in the mean being 0% increases the more times the values are 0%, including across individuals (e.g., it would be highly unlikely that 30 fish in a row spend 0.4% of their time in predator cue; chance would have some of them fall over to 0.6% and thus give a few 1% recordings). It is therefore incorrect to use 1% as an estimate for a mean of 0% (and similarly incorrect to use a proportion of 0.01 to estimate a mean proportion of 0), given the number of times 0% was recorded without any variance around that mean. Using the reasoning for precision estimates above, we estimated mean percentages to four decimal places in our original analysis [1]. While meta-analysis best practices are not available for such data, and we can appreciate the position that 0.0001 may overestimate the precision of the measurements, using 1% is nonetheless incorrect. Munday’s use of 0.1% to estimate 0% (0.001 for proportional data) in his Fig 1 in [2], is reasonable and still depicts an extreme decline effect for this field. It is important to note here, however, that the actual means and variances reported in these studies are *precisely* zero and that such means and variance are highly unlikely for biological data—particularly for something as inherently variable as animal behavior. Our initially reported decline effect, therefore, is not simply a statistical artifact but represents the extreme differences between treatment means, and the highly improbable variances around those means, in early studies. The choice of values to represent 0% means simply dictates the magnitude of extremeness.

Further examples of means of 0% and 100%, with extremely small variances, are present in other papers led by Dixson and Munday (e.g., [6,7]). Using bootstrapping simulations (see Supp. Appendix at https://osf.io/7spzx/ for details), with choice-flume behavioral data extracted from videos available online from Munday’s lab [8], we demonstrate that such low variances are highly improbable and likely nonbiological (**Fig 1A–1C**). These extreme data, and the methods used to obtain them, are consistent with results in three publications by Dixson that were found to contain fabricated data [4,9]. Among these is a paper in *Science* that has been retracted for data fabrication [10]. Thus, while Munday argues that our initial effect size estimates are artificially “extreme,” the fact remains that those effect sizes are derived from extreme and implausible initial data. Indeed, as concluded in our original analysis, the factor with the highest explanatory power for the decline effect is still the influence of “authors,” specifically Dixson and/or Munday (**Fig 1D and 1E**).

**Fig 1:**
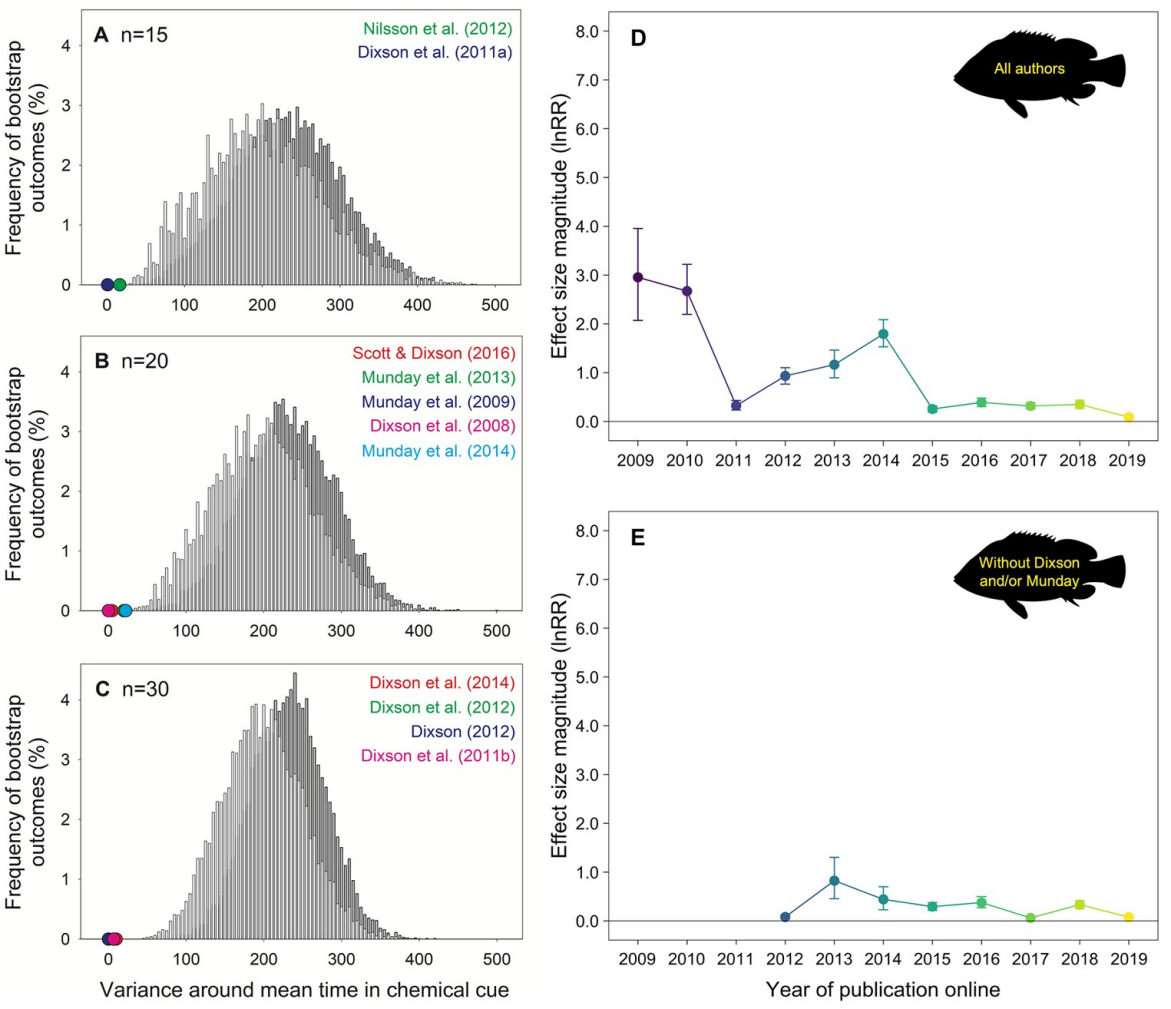
**(A–C)** Distributions (vertical bars) of the probability of obtaining a particular variance around a mean value of “percent time in chemical alarm cue” when examining groups of juvenile spiny chromis (*Acanthochromis polyacanthus*) with sample sizes of 15 **(A)**, 20 **(B)**, and 30 **(C)**. Distributions were produced from 10,000 bootstrapping simulations per panel when using the control (white bars) and high CO_2_ (grey bars) data obtained from behavioral videos from Munday’s lab (see Table A1 in Supp. Appendix) and defining the stated sample sizes. Sample sizes were selected to match those reported in previous papers by Munday and Dixson (colored circles from a subset of references noted in each panel), where a range of species have been tested using various chemical cues (not all associated with ocean acidification). Circle symbols are sometimes hidden behind each other. Note that all papers by Munday and Dixson contain data with variances that are lower than plausible based on the available video evidence. Full references for the papers identified in the figure panels can be found in Supp. Appendix. (**D**, **E**) Mean effect size magnitude (absolute lnRR ± upper and lower confidence bounds) for each year of publication online estimated from our revised dataset (i.e., after including or excluding Munday’s data “corrections”; see Supp. Data 1) including all studies **(D)** and the dataset excluding studies authored or coauthored by Dixson and/or Munday (all papers before 2012 were authored by Dixson and/or Munday) **(E)**. Mean effect size magnitudes and confidence bounds were estimated using Bayesian simulations and a folded normal distribution. Colors in panels D and E are aesthetic in nature and follow a gradient according to year of publication. Effect size magnitudes and their uncertainty were estimated from our revised dataset using the same analytical approach as detailed in [1]. Annotated R code and raw data files for the meta-analysis in panels D and E are in Supp. Code, Supp. Data 2, and Supp. Data 3. Panels A–C were created using SigmaPlot Version 11.0 (Systat Software, San Jose, CA), while panels D and E were created using R (*ggplot2* package; [13]). Underlying data for each figure panel can be found in Supp. Data 2. All supplementary files can be found at https://osf.io/7spzx/.

The rhetoric of Munday [2] implies that there is no decline effect in this field, but Munday’s real argument is that the decline effect is not “extreme,” as stated in [1]. However, the decline effect in Munday’s reanalysis is still numerically extreme; a decline in mean effect size magnitude (lnRR) from ≈3 to <0.5 (see Fig 1A in [2]) is a very large decline. For example, in [5], a lnRR of 3.3 is derived from control and OA-exposed fish spending 3% and 85% of their time in predator cues, respectively, while a lnRR of 0.4 comes from control and OA-exposed fish, respectively, spending 12% and 18% of their time in predator cues. That is, to achieve lnRR ≈3.3 (approximate mean lnRR of early studies using revised values for zero means), there is a difference of 82% in the time spent in a predator cue between control and OA-exposed fish, while lnRR ≈0.4 (approximate revised mean lnRR for later studies) results from a difference of only 6%. To put this in the context of other decline effects in ecology and evolution, Jennions and Møller [3] reported that the strongest decline effects in their dataset of ecological meta-analyses had Z-transformed Spearman correlation coefficients for the relationship between year and mean effect size between −0.5 and −0.7 (*n* = 4 of 44 studies; see Fig 1 in [3]). Comparatively, the Z-transformed Spearman correlation in Munday’s reanalysis is −0.679 (computed using the data underlying Fig 1D in [2]; see Supp. Data 3 at https://osf.io/7spzx/), compared to a Z-transformed Spearman correlation of −0.904 in our original analysis [1]. Similarly, the Pearson correlation for the relationship between year and mean effect size in Munday’s reanalysis is −0.697 (−0.711 in our original analysis)—a negative Pearson correlation exceeded by only 3 out of 466 ecological meta-analyses identified in a recent meta-meta-analysis of decline effects from Costello and Fox ([11]; see Fig 2A therein]). Thus, the decline effect identified in Munday’s reanalysis remains one of the most extreme in ecology and evolution.

We are thrilled to see that sharing our raw data and code has led to reanalysis of our original dataset—a testament to the value of open science [12]. We are also thankful that a few errors in data extraction were identified, so that they could be corrected. Notably, Munday’s reanalysis shows that these errors did not alter the main findings of our work despite the rhetoric of his comment implying no decline effect.

Whether one calls it “extreme,” “strong,” or without adjective, the field of ocean acidification and fish behavior constitutes a clear, textbook example of the decline effect that is most parsimoniously explained by the authors of the papers included in the meta-analysis. Our analyses ([1]; **Fig 1D and 1E**) and Munday’s reanalysis [2] all support this conclusion.

## References

[1] Clements JC, Sundin J, Clark TD, Jutfelt F. Meta-analysis reveals an extreme “decline effect” in the impacts of ocean acidification on fish behavior. PLoS Biol. 2022;20(2):e3001511. doi: 10.1371/journal.pbio.3001511 35113875 PMC8812914

[2] Munday PL. Reanalysis shows the extreme decline effect does not exist in fish ocean acidification studies. PLoS Biol. 2022;20(11):e3001809. doi: 10.1371/journal.pbio.3001809 36413526 PMC9681065

[3] Jennions MD, Møller AP. Relationships fade with time: a meta-analysis of temporal trends in publication in ecology and evolution. Proc R Soc B. 2002;269:43–48. doi: 10.1098/rspb.2001.1832 11788035 PMC1690867

[4] Enserink M. Sea of doubts: Dozens of papers linking high carbon dioxide to unsettling changes in fish behavior fall under suspicion. Science. 2021;372:560–565. doi: 10.1126/science.372.6542.560 33958459

[5] Dixson DL, Munday PL, Jones GP. Ocean acidification disrupts the innate ability of fish to detect predator olfactory cues. Ecol Lett. 2010;13:68–75. doi: 10.1111/j.1461-0248.2009.01400.x 19917053

[6] Munday PL, Dixson DL, Donelson JM, Jones GP, Pratchett MS, Devitsina GV, et al. Ocean acidification impairs olfactory discrimination and homing ability of a marine fish. Proc Natl Acad Sci U S A. 2009;106:1848–1852. doi: 10.1073/pnas.0809996106 19188596 PMC2644126

[7] Munday PL, Dixson DL, McCormick MI, Meekan M, Ferrari MCO, Chivers DP. Replenishment of fish populations is threatened by ocean acidification. Proc Natl Acad Sci USA. 2010;107:12930–12934. doi: 10.1073/pnas.1004519107 20615968 PMC2919925

[8] Welch M, Munday PL. Raw data for olfactory response of *Acanthochromis polyacanthus* in a Y-maze flume (dataset). Database: Research Data JCU [Internet]. 2013. doi: 10.4225/28/5add60af3a267

[9] Enserink M. Star marine ecologist guilty of misconduct, university says. Science. 2022;377:699–700. doi: 10.1126/science.ade3374 35951688

[10] Thorp HH. Editorial retraction. Science. 2022;377:826. doi: 10.1126/science.ade2691 35943365 PMC9446476

[11] Costello L, Fox JW. Decline effects are rare in ecology. Ecology. 2022;103:e3680. doi: 10.1002/ecy.3680 35302660

[12] Roche DG, Raby GD, Norin T, Ern R, Scheuffele H, Skeeles M, et al. Paths towards greater consensus building in experimental biology. J Exp Biol. 2022;225:jeb243559. doi: 10.1242/jeb.243559 35258604

[13] Wickham H. ggplot2: Elegant Graphics for Data Analysis. New York: Springer-Verlag; 2016.

